# Resistance Characterization of *Plasmopara viticola* to Metalaxyl, Cymoxanil, and Cyazofamid in China

**DOI:** 10.3390/jof12030180

**Published:** 2026-03-03

**Authors:** Meng-Zhen Yang, Lian-Zhu Zhou, Fan-Fang Kong, Shao-Wei Cui, Yong-Qiang Liu, Zhong-Yue Wang, Shi-Dong Li, Rong-Jun Guo, Kang Qiao, Xiao-Qing Huang

**Affiliations:** 1 College of Plant Protection, Shandong Agricultural University, Taian 271018, China; yangmz0204@163.com; 2 State Key Laboratory for Biology of Plant Diseases and Insect Pests, Institute of Plant Protection, Chinese Academy of Agricultural Sciences, Beijing 100193, China; pillar1017@126.com (L.-Z.Z.); fanfang103@126.com (F.-F.K.); shaowei_cui@126.com (S.-W.C.); liuyongqiang@caas.cn (Y.-Q.L.); wangzhongyue0301@163.com (Z.-Y.W.); sdli@ippcaas.cn (S.-D.L.); guorj20150620@126.com (R.-J.G.)

**Keywords:** *Plasmopara viticola*, metalaxyl, cymoxanil, cyazofamid, fungicide resistance, fitness

## Abstract

Downy mildew, caused by *Plasmopara viticola*, is a devastating disease that threatens global grape production, with chemical control remaining the most effective management strategy. However, the repeated application of fungicides has led to widespread resistance in *P. viticola* populations, while data on the resistance of *P. viticola* to metalaxyl (MET), cymoxanil (CYM), and cyazofamid (CYA) in China remain limited. In this study, the resistance status of *P. viticola* to these three fungicides was evaluated across 9 major grape-growing regions in China using leaf-disc bioassays, and potential cross- and multi-resistance patterns were assessed. The majority of isolates (127/233) exhibited either lower resistance (33.48%) or moderate resistance (21.03%) to MET based on the minimum inhibitory concentration (MIC) of 10 μg/mL and 100 μg/mL. Baseline sensitivity profiles for CYM and CYA were established as 8.69 ± 0.64 μg/mL and 0.42 ± 0.05 μg/mL, respectively, using 170 and 137 isolates. The total resistance frequency of *P. viticola* to CYM was 29.42% (21.18% low resistance, 8.24% moderate resistance), while that to CYA was 28.47% (18.25% low resistance, 9.49% moderate resistance, 0.73% high resistance). A weak but significant positive correlation was detected between CYM and CYA sensitivities (*r* = 0.193, *p* = 0.0196), and 13 isolates exhibited resistance to both fungicides, indicating potential multi-resistance risk. Significant regional differences in resistance profiles were observed among populations (*p* < 0.05), and no overall fitness penalties were detected. These findings highlight the necessity of region-specific and integrated resistance management strategies for sustainable control of grape downy mildew in China.

## 1. Introduction

Grape (*Vitis vinifera* L.) is one of the oldest cultivated fruit crops globally, widely grown due to its high consumption and substantial economic value [1]. As a major grape-producing country, China plays a crucial role in the global viticulture industry. In 2022, China’s vineyard area reached 705, 113 hectares with an annual output of 15.38 million metric tons, making grape cultivation a vital component of the national agricultural economy and rural development [2].

Downy mildew, caused by *Plasmopara viticola*, is one of the most devastating diseases affecting grapevines worldwide, severely threatening grape production [3]. Originally from eastern North America, the pathogen was introduced to Europe in the 1870s and has since become a persistent global challenge for viticulture [4]. In China, *P. viticola* was first reported in the 1980s and has since posed a serious threat, particularly in major grape-growing regions [5]. Most commercial grape cultivars remain highly susceptible to this pathogen, making disease control critical for sustainable viticulture. The management of grapevine downy mildew primarily relies on integrated strategies, including the cultivation of resistant varieties, agronomic practices, biological control, and chemical applications. Although progress has been made in breeding and cultural management, chemical control remains the most effective and widely adopted strategy—especially under favourable environmental conditions for disease outbreaks [6]. Several systemic fungicides have been proven effective against *P. viticola*, including Carboxylic Acid Amide (CAA) fungicides, Quinone outside inhibitor (QoIs) fungicides, metalaxyl (MET), cymoxanil (CYM), and cyazofamid (CYA) [7,8]. However, the resistance of CAA and QoI fungicides has become relatively widespread, particularly in China [9,10], while limited research has been conducted on *P. viticola* resistance to MET, CYM, and CYA.

Metalaxyl (MET), the first phenylamide fungicide introduced in 1977, revolutionized oomycete disease management [11]. As a systemic fungicide, it is readily absorbed and interferes with RNA polymerase I, thereby inhibiting rRNA synthesis [12]. Cymoxanil (CYM), a synthetic acetamide fungicide also introduced in the late 1970s, is mainly used as a foliar spray and exhibits efficacy against *Phytophthora infestans*, tomato blight, and grapevine downy mildew [13,14,15]. Despite a long and extensive history of application, the precise mode of action of CYM remains unknown [16]. According to some authors, CYM acts as a pro-fungicide and undergoes biotransformation by fungi into one (or several) fungitoxic metabolite(s) [17,18]. CYM seems to affect several biochemical processes, such as the synthesis of nucleic acids and/or amino acids in *Botrytis cinerea* Pers [18]. Cyazofamid (CYA), a relatively new fungicide, demonstrates strong efficacy against grape downy mildew and other oomycete diseases, including potato powdery scab, *P. infestans*, and *Phytophthora* root rot and wilt [19,20,21,22]. It acts by selectively binding to cytochrome b, disrupting mitochondrial electron transport in complex III [23].

The repeated and widespread use of fungicides has led to the development of resistance in numerous pathogen populations. Resistance to MET was first detected in *P. viticola* populations in France in 1981 [24,25] and has since spread to most Atlantic coastal regions with frequent fungicide use [26]. Long-term monitoring data from countries with systematic sensitivity testing (e.g., France, Switzerland, Spain, Germany) showed that the proportion of resistant isolates has stabilized at a high level (50–80%) for an extended period [27]. Continuous sensitivity surveillance, including data from 2017/2018, has validated the persistence of this resistance profile [28]. The resistance mechanism may involve one (or two) major gene(s) and potentially several minor genes. The target gene and the site of mutation(s) in the genome have not been mapped so far [28]. Resistance to CYM was first reported in *P. viticola* populations in northern Italy in the 1990s [29] and has since been documented across Europe and India [30,31,32]. In contrast, reports on *P. viticola* resistance to CYA remain scarce. Early studies focused on *Phytophthora capsica* [33,34], but recent studies have detected *P. viticola* populations with reduced sensitivity to CYA in French vineyards [35,36]. Moreover, it has been found that the resistance of these strains may be associated with a point mutation (L201S) and insertions (E203-DE-V204, E203-VE-V204) in the CYTB gene [36].

Despite the global prevalence of fungicide resistance, data regarding the resistance of *P. viticola* to MET, CYM, and CYA remain scarce in China. Elucidating the current resistance status of this pathogen is therefore critical to optimizing the management of grape downy mildew. This study aims to evaluate the resistance status of *P. viticola* to MET, CYM, and CYA in major grape-growing regions of China. The specific objectives are as follows: (1) to assess the sensitivity of isolates to the three fungicides using the leaf disc assay, and to establish baseline sensitivity profiles for CYM and CYA for evaluating the potential risk of resistance evolution; (2) to determine the frequency and geographic distribution of fungicide-resistant populations; (3) to compare the fitness of wild-type and resistant isolates by analyzing key biological traits (infection frequency, lesion area, and sporulation capacity). The Findings will contribute to formulating effective fungicide resistance management strategies and provide a scientific basis for the sustainable control of grape downy mildew in China.

## 2. Materials and Methods

### 2.1. Fungicides

Metalaxyl (MET, 87% active ingredient [a.i.]), cymoxanil (CYM, 98.2% a.i.), and cyazofamid (CYA, 95% a.i.) were provided by the Institute of Plant Protection, Chinese Academy of Agricultural Sciences (CAAS), Beijing, China. MET was dissolved in methanol to prepare a 50 mg/mL stock solution. CYM (0.1018 g) was dissolved in 10 mL of methanol to prepare a 10 mg/mL stock solution. CYA (0.1053 g) was dissolved in 10 mL of acetone to prepare a 10 mg/mL stock solution. All stock solutions were stored at 4 °C for subsequent use.

### 2.2. Isolates

During the 2017–2018 growing seasons, *P. viticola* samples were collected from 9 grape-producing regions across China. Isolation and purification were performed using the single-sporangiophore method described by Zhang et al. [37]. Briefly, infected leaves were placed in Petri dishes lined with moist filter paper, sprayed with sterile water, and incubated in an artificial climate chamber (21 °C, 16 h light/8 h dark photoperiod) to induce sporulation. A single sporangiophore was isolated under a stereomicroscope and transferred to the abaxial surface of sterilized grape leaf discs (15 mm diameter) placed on 1% water agar. After 24 h of dark incubation at 21 °C, excess moisture was removed, and discs were incubated under the same light/dark cycle for 6 days until dense sporulation appeared. Sporangia were harvested, suspended in sterile deionized water, and adjusted to 1 × 10^5^ sporangia/mL to inoculate new leaf discs for pure isolate propagation. A total of 233 single-sporangiophore *P. viticola* isolates were obtained (Table 1). Among these, 233 were tested for MET sensitivity, 170 for CYM sensitivity, and 137 for CYA sensitivity. Leaf discs with single sporangia and sporangiophores were collected and stored in liquid nitrogen.

### 2.3. Sensitivity Assay

#### 2.3.1. Metalaxyl (MET)

The sensitivity of *P. viticola* to MET was determined by using the leaf disc method. Discriminatory concentrations of 10 μg/mL and 100 μg/mL were selected based on published baseline sensitivity distributions and resistance monitoring protocols, ensuring consistency with established surveillance studies and enabling meaningful comparison across datasets [38,39,40]. Leaf discs (15 mm diameter) from *V. vinifera* cv. Rizamat seedlings (3rd–5th leaves from the shoot tip) were floated abaxially up on fungicide solutions in beakers. Leaf discs treated with sterile water served as controls. After 24 h, discs were transferred to RAP agar medium (1.5% agar, 30 mg/mL rifampicin, 150 mg/mL sodium ampicillin, 5 mg/mL pimaricin) and inoculated with 10 μL of sporangial suspension (1 × 10^5^ sporangia/mL). Each treatment included 10 leaf discs with three replicates. Lesion incidence was visually assessed at 7 days post-inoculation (dpi). Isolates were classified as: (1) sensitive (S): no pathogenicity at 10 μg/mL (control showed disease); (2) low-level resistant (LR): pathogenicity at 10 μg/mL but not at 100 μg/mL (resistance factor [RF] = 100); (3) resistant (R): pathogenicity at 100 μg/mL (RF ≥ 1000) [38,41].

#### 2.3.2. Cymoxanil (CYM) and Cyazofamid (CYA)

For CYM and CYA, the sensitivity of *P. viticola* was also determined by using the leaf disc method [38]. Five concentrations were tested for each fungicide: 1, 10, 20, 40, 60 μg/mL for CYM; 0.1, 0.5, 2, 8, 16 μg/mL for CYA. Sterile water plus solvent served as controls. Leaf disc preparation and incubation followed the procedures described above. Disease severity was rated at 7 dpi using a 0–9 scale [42]: 0 = no lesions; 1 = lesion area < 5%; 3 = 6–25%; 5 = 26–50%; 7 = 51–75%; 9 = >75%.

The disease index (DI) was calculated as:

DI = 100 × ∑(Number of leaf discs at each grade × Grade value)/(Total leaf discs assessed × Maximum grade value)

The inhibition rate (%) was determined by:

Inhibition rate = 100 × (DIcontrol − DItreatment)/DI control

EC_50_ values (effective concentration inhibiting 50% of disease development) were calculated using probit analysis. Baseline sensitivity was defined as the mean EC_50_ value of isolates with a unimodal EC_50_ frequency distribution [43]. Resistance factors (RFs) were calculated as the ratio of the EC_50_ of a test isolate to the mean EC_50_ of the baseline population. Isolates were classified based on RF values [44]: sensitive (S, RF ≤ 3), low resistance (LR, 3 < RF ≤ 10), moderate resistance (MR, 10 < RF < 100), and high resistance (HR, RF ≥ 100).

### 2.4. Fitness Determination

According to the method established by Liu et al. [45], the fitness of resistant and sensitive isolates was evaluated by assaying three key traits: infection frequency, lesion area, and sporulation capacity. Tested isolates were randomly selected. Resistant isolates were selected to represent the resistance spectrum observed in the population. Specifically, MET-resistant (R) isolates included 63 strains (34 LR and 29 MR) and were compared with 96 MET-sensitive (S) isolates. For CYM, 13 resistant isolates (5 LR and 8 MR) and 15 sensitive isolates were tested. For CYA, 11 resistant isolates (5 LR, 5 MR and 1 HR) and 13 sensitive isolates were included. No highly resistant isolates were available for MET and CYM. LR MR and HR isolates were analyzed collectively as R for fitness comparison. Leaf discs were inoculated with 20 μL of sporangial suspension (1 × 10^5^ sporangia/mL) and incubated as described in Section 2.3. At 7 dpi, the following parameters were measured:

Infection frequency (%): (number of infected discs/Total number of discs) × 100

Lesion area (cm^2^/leaf disc): Average diseased area per isolate

For the determination of sporulation capacity, 1 mL of sterile water was used to rinse the sporangia from the sporangia-producing leaf discs into a centrifuge tube. The sporangial suspension was adjusted to a final volume of 1 mL, shaken thoroughly to homogenize, and 10 μL of the suspension was aspirated with a pipette and loaded onto a hemocytometer. Sporangia were counted under a light microscope, with three replicates performed for each isolate. Sporulation capacity (SC) was defined as the number of sporangia produced per unit volume (mL) per unit leaf disc area (cm^2^).

Subsequently, the overall fitness of resistant and sensitive *P. viticola* isolates to the three fungicides was evaluated using a composite fitness index (CFI), calculated as:

CFI = Disease incidence × Lesion area × Sporulation capacity

### 2.5. Data Analysis

To quantify and compare the resistance differences among *P. viticola* populations from different regions, two evaluation indices were established in this study: the methodology described by Zhou [46] was used to calculate the Resistance Frequency (RFr) and Resistance Level Index (RLI), with some modifications. RFr was defined as the proportion of resistant isolates in the population, categorized into three grades: low (<20%), moderate (20~60%), and high (>60%). To assess the overall resistance level of each population, strain phenotypes were converted into corresponding numerical values (susceptible = 0, low resistance = 1, moderate resistance = 2, high resistance = 3), and the weighted RLI was calculated using the formula: RLI = 100 × ∑(resistance level value × number of strains at that level)/(maximum resistance level value × total number of strains). The grading standard for RLI was consistent with that of RFr (low < 20, moderate 20~60, high > 60). Furthermore, to measure cross-resistance between CYM and CYA, Pearson correlation coefficients (*r*) between EC_50_ values of different isolates under different treatments were calculated.

Differences in resistance profiles among regions were quantified using the Bray–Curtis distance matrix, and principal coordinate analysis (PCoA) was performed to visualize regional variations in resistance characteristics. All resistance-related statistical analyses were conducted in R software (v4.4.2) using the *vegan* package (2.6-8): pairwise Adonis analysis was applied to test the significance of inter-regional resistance differences, and permutational multivariate analysis of variance (PERMANOVA) with 999 permutations was used to determine the contributions of intra-regional isolate resistance frequency and level to inter-regional variations in resistance profiles.

Independent samples *t*-tests and one-way analysis of variance (ANOVA) were also conducted in R software (v4.4.2) to compare fitness-related parameters between resistant and sensitive isolates, with Fisher’s protected least significant difference (LSD) test for post hoc comparisons. Differences were considered significant at *p* < 0.05.

## 3. Results

### 3.1. Baseline Sensitivity of P. viticola to Cymoxanil and Cyazofamid

**Figure 1:**
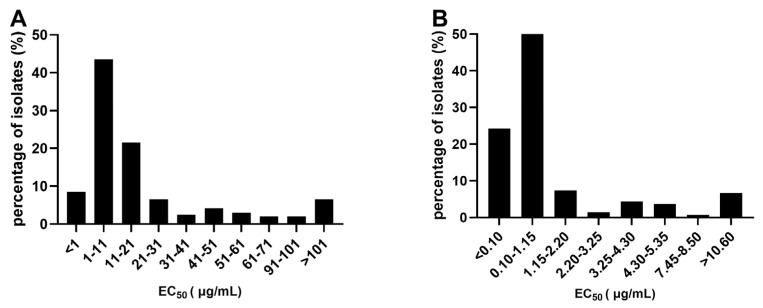
Frequency distributions of 50% effective concentrations (EC_50_ values) for (**A**) cymoxanil (CYM) and (**B**) cyazofamid (CYA) against *P. viticola* isolates collected from vineyards in China (based on leaf disc assays).

The sensitivity of *P. viticola* to cymoxanil (CYM) and cyazofamid (CYA) was tested by leaf disc assay. The EC_50_ values of all isolates to CYM ranged from 0.01 to 691.39 μg/mL, with a mean value of 36.44 μg/mL and were separated by a factor of 69,139 (Table 2). Regardless of the relatively wide EC_50_ range, most EC_50_ values (79.4%) were below 31 μg/mL, with a mean value of 8.69 ± 0.64 μg/mL, and their frequency distribution presented a unimodal curve (Figure 1A), which could be used as the baseline sensitivity of *P. viticola* to CYM. The EC50 values for CYA ranged from 0.01 to 60.39 μg/mL and thus were separated by a factor of 6039 (Table 2). A total of 113 isolates (82.5%) showed EC_50_ values lower than 2.2 μg/mL, and the frequency distribution of the EC_50_ values showed a unimodal curve with a mean value of 0.42 ± 0.05 μg/mL (Figure 1B), which could be used as the baseline sensitivity of *P. viticola* to CYA.

### 3.2. Resistance Characteristics of P. viticola to Metalaxyl, Cymoxanil, and Cyazofamid

**Figure 2:**
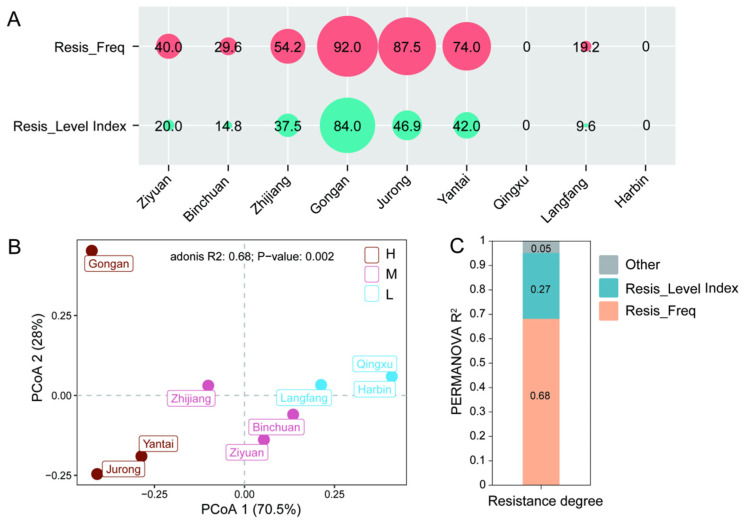
Differences in resistance grades of *P. viticola* to metalaxyl (MET) among nine regions. (**A**) Resistance frequency and resistance level values of the isolates from nine regions. Note: Resis_Freq = Resistance frequency, Resis_Level Index = Resistance level index; (**B**) Principal coordinate analysis (PCoA) of resistance grades among nine regions based on Bray–Curtis distance (*n* = 9), where H, M and L represent high, medium and low resistance grades, respectively; (**C**) Contribution of resistance frequency and resistance level to the differences in resistance grades among regions was evaluated by permutational multivariate analysis of variance (PERMANOVA).

The resistance frequency and resistance levels of *P. viticola* isolates collected from different regions to MET, CYM and CYA were determined based on the minimum inhibitory concentration (MIC) of MET and the median effective concentration (EC_50_) of CYM and CYA. Of 233 tested *P. viticola* isolates, 45.49% were sensitive to MET, with a total resistance frequency of 54.51% (33.48% low-level resistance, 21.03% resistance). Overall, the isolates showed relatively low-level resistance, predominantly sensitive and low-level resistant types (Table S1). PCoA based on the Bray–Curtis distance revealed significant spatial structural differences in the resistance of *P. viticola* populations to MET among the nine regions (Figure 2). This analysis further identified two core factors driving inter-regional variations: the first principal coordinate axis (PCoA1) primarily reflected differences in the resistance frequency (RFr) of pathogen populations, while the second principal coordinate axis (PCoA2) represented differences in resistance levels. These results indicated that populations with high resistance frequency (Gong’an, Jurong and Yantai) were significantly separated from those with moderate resistance (Ziyuan, Binchuan, and Zhijiang) and low resistance (Qingxu, Harbin, and Langfang), suggesting that the prevalence of resistant isolates is the primary factor distinguishing inter-regional resistance differences. In addition, although Gong’an and Jurong exhibited similar resistance frequencies, they were still significantly separated along PCoA2, indicating that there were also statistical differences in their resistance levels (Figure 2B). PERMANOVA (adonis) statistical analysis further validated these observations, confirming that the resistance profiles among different regions exhibited significant differences (*p* < 0.05). The variance partitioning results showed that resistance frequency contributed the most to the total variation, followed by resistance level (Figure 2C).

**Figure 3:**
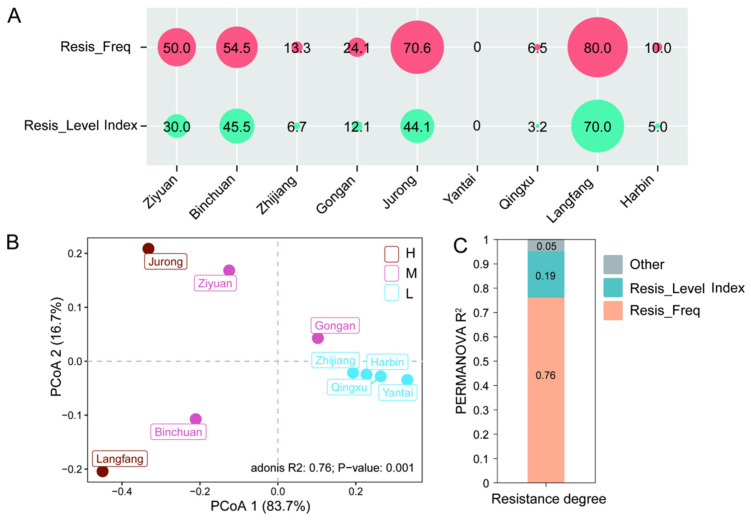
Differences in resistance grades of *P. viticola* to cymoxanil (CYM) among nine regions. (**A**) Resistance frequency and resistance level values of the isolates from nine regions. Note: Resis_Freq = Resistance frequency, Resis_Level Index = Resistance level index; (**B**) Principal coordinate analysis (PCoA) of resistance grades among nine regions based on Bray–Curtis distance (*n* = 9), where H, M and L represent high, medium and low resistance grades, respectively; (**C**) Contribution of resistance frequency and resistance level to the differences in resistance grades among regions was evaluated by permutational multivariate analysis of variance (PERMANOVA).

For CYM resistance, 70.59% (120/170) of the isolates were sensitive, 21.18% (36/170) exhibited low resistance, and 8.24% (14/170) exhibited moderate resistance; no isolates met the criteria for high resistance at the individual level (Table S1). PCA analysis revealed significant differences in the CYM-resistance profiles among *P. viticola* populations from different regions. Based on population-level resistance metrics, the entire pathogen population could be clustered into three distinct subgroups reflecting relative resistance status across regions. Strains from Jurong and Langfang were classified into the high-resistance subgroup, those from Binchuan, Ziyuan, and Gong’an belonged to the moderate-resistance subgroup, and strains from Zhijiang, Harbin, Yantai, and Qingxu were assigned to the low-resistance subgroup (Figure 3B). Further analysis indicated that resistance frequency was the most prominent factor influencing the overall CYM resistance of the population (Figure 3C). Notably, some regions (e.g., Langfang and Jurong) exhibited a severe resistance pattern characterized by high resistance frequency and level. The results indicated that there were significant differences in CYM resistance among *P. viticola* populations from different regions, with some regions exhibiting a severe resistance pattern characterized by both high resistance frequency and high resistance level.

**Figure 4:**
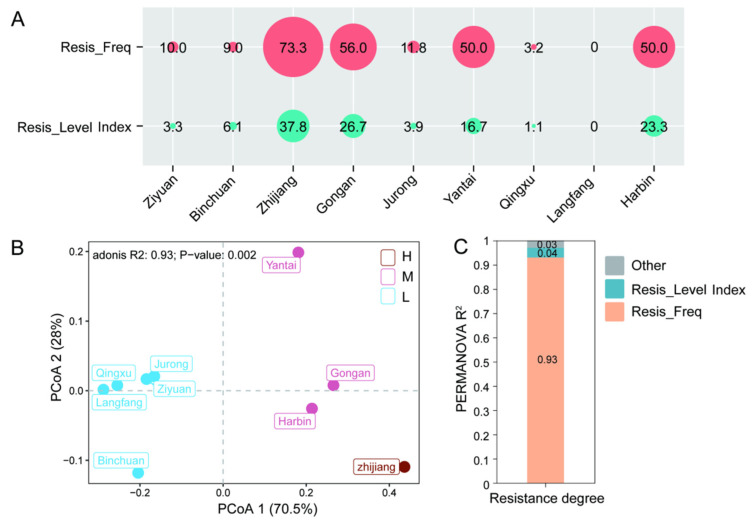
Differences in resistance grades of *P. viticola* to cyazofamid (CYA) among nine regions. (**A**) Resistance frequency and resistance level values of the isolates from nine regions. Note: Resis_Freq = Resistance frequency, Resis_Level Index = Resistance level index; (**B**) Principal coordinate analysis (PCoA) of resistance grades among nine regions based on Bray–Curtis distance (*n* = 9), where H, M and L represent high, medium and low resistance grades, respectively; (**C**) Contribution of resistance frequency and resistance level to the differences in resistance grades among regions was evaluated by permutational multivariate analysis of variance (PERMANOVA).

For CYA resistance (137 isolates tested), 71.53% (98/137) were sensitive, 18.25% (25/137) were low-resistant, 9.49% (13/137) were moderate-resistant, and 0.73% (1/137) were high-resistant (Table S1). Significant regional differences in the CYA-resistance profiles were also confirmed by PCA analysis (Figure 4B). The entire pathogen population could be clustered into three distinct subgroups: strains from Zhijiang were classified into the high-resistance subgroup, those from Gong’an, Harbin, and Yantai belonged to the moderate-resistance subgroup, and strains from Jurong, Ziyuan, Binchuan, Langfang, and Qingxu were assigned to the low-resistance subgroup (Figure 4B). Overall, the resistance level of the pathogen population was relatively low, and resistance frequency exerted the most significant contribution to the overall resistance of the population. These observations were further validated by PERMANOVA (adonis) statistical analysis, confirming that inter-regional differences in CYA resistance were statistically significant and that such variations were primarily attributed to differences in resistance frequency (Figure 4C).

### 3.3. Cross- and Multi-Resistance Patterns

**Figure 5:**
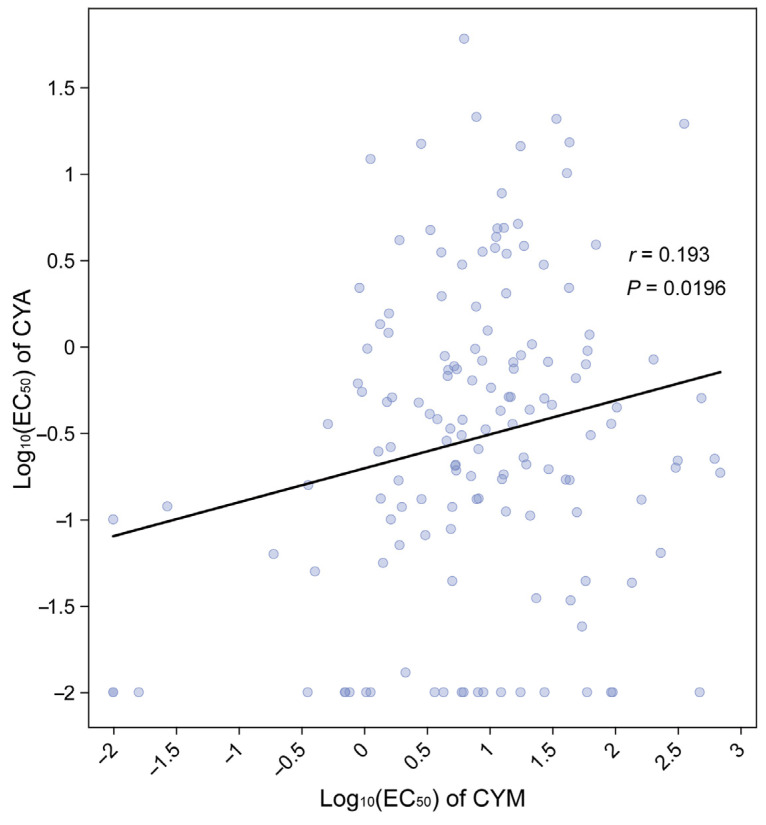
Relationship between CYM and CYA sensitivity in *P. viticola* isolates. Log_10_-transformed EC_50_ values of CYM were plotted against Log_10_-transformed EC_50_ values of CYA. Each point represents an individual isolate.

To clarify whether there is cross-resistance between CYM and CYA, log-transformed EC_50_ values were analyzed by establishing correlation and linear regression. The Pearson correlation test revealed there was a weakly positive correlation between resistance to CYM and to CYA (r = 0.193, *p*= 0.0196; Figure 5). Furthermore, there are 13 isolates exhibit resistance to both CYM and CYA (Table S2).

### 3.4. Fitness of P. viticola to Metalaxyl, Cymoxanil, and Cyazofamid

**Figure 6:**
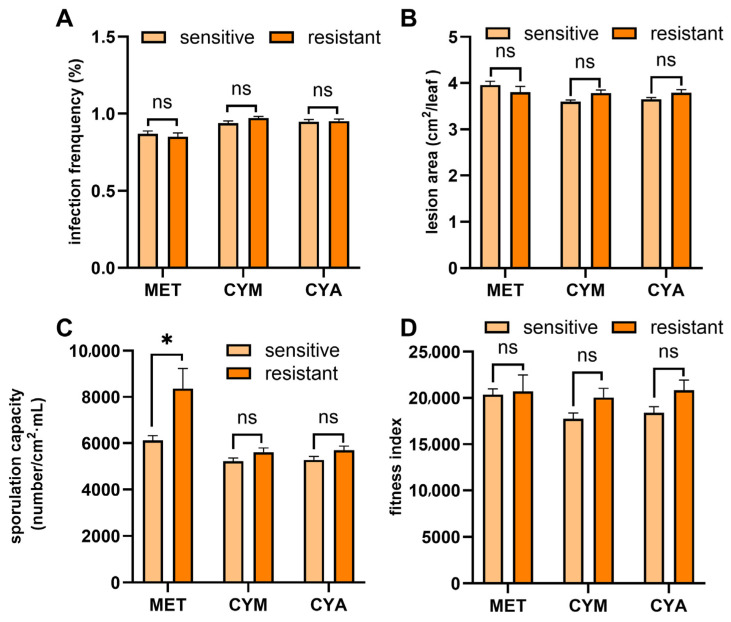
Fitness comparison between fungicide-resistant and sensitive isolates of *P. viticola* to metalaxyl (MET), cymoxanil (CYM) and cyazofamid (CYA). (**A**) Infection frequency; (**B**) Lesion area; (**C**) Sporulation capacity; (**D**) Fitness index. * indicates a significant difference (*p* < 0.05), and ns indicates no significant difference (*p* ≥ 0.05).

For MET, the sporulation capacity of resistant strains was significantly higher than that of sensitive strains (*p* < 0.05, Figure 6C), while no significant differences were observed in infectivity, lesion area, or composite fitness index between the two strain types (Figure 6A,B,D). In contrast, resistant and sensitive strains showed no significant variations in all the aforementioned fitness-related traits for CYM and CYA (Figure 6). Collectively, these results indicate that the fitness of resistant *P. viticola* to the three fungicides did not change significantly.

## 4. Discussion

This study systematically investigated *P. viticola* resistance to metalaxyl (MET), cymoxanil (CYM), and cyazofamid (CYA) in 9 major Chinese grape-producing regions, established CYM/CYA sensitivity baselines and compared resistant/sensitive strain fitness. The results provide a critical scientific basis for downy mildew chemical control and resistance management.

The overall MET resistance frequency of *P. viticola* in China was 54.51% (33.48% low-level, 21.03% high-level), which aligns with the global evolutionary trend of MET resistance. In recent years, sensitivity analyses conducted in countries including France, Switzerland, Spain, and Germany have confirmed that the proportion of MET-resistant *P. viticola* isolates remains high (50–80%) and relatively stable [27]. Sensitivity monitoring data from 2017/2018 (consistent with the sampling period of this study) further confirmed this global resistance pattern [28]. The relatively high MET resistance frequency observed in China may be associated with the early extensive use of MET for downy mildew control. Significant regional differences in MET resistance among *P.viticola* populations were observed in China (*p* < 0.05). For instance, strains from Gong’an and Jurong exhibited high resistance frequency and high resistance level, while those from Qingxu, Harbin, and Langfang showed extremely low resistance. Notably, all strains from Qingxu and Harbin were sensitive. This is likely related to local downy mildew incidence and fungicide use. Severe MET resistance is mainly distributed in central and southern China, where rainy and humid conditions cause severe downy mildew outbreaks and higher fungicide application frequencies—presumably the primary driver of high pathogen resistance.

Based on the established sensitivity baseline of CYM against *P. viticola* (8.69 ± 0.64 μg/mL, EC_50_), our analysis of isolates from 9 Chinese grape-growing regions revealed that 70.59% remained sensitive to CYM, with resistant isolates mainly being low-resistance biotypes. This resistance profile aligns with Toffolatti et al. [47], who reported a low proportion of CYM-resistant *P. viticola* populations in Italy. This consistency is presumably attributed to the common field application of CYM as mixed formulations, which have been proven to delay pathogen resistance evolution [31]. Notably, distinct resistance differentiation was observed across regions. Isolates from Langfang and Jurong showed high resistance frequencies (80% and 70.6%, respectively) and considerable moderate-resistance proportions (60% and 17.6%), indicating a high CYM resistance risk in these areas. This phenomenon is likely associated with local intensive fungicide use, emphasizing the necessity of targeted management strategies. For regions with severe resistance, strict restrictions on CYM application are imperative. In areas with moderate resistance, CYM should be applied as a mixed formulation component or rotated with fungicides of different modes of action to mitigate selection pressure and delay resistance development.

To the best of our knowledge, this is the first study to establish the sensitivity baseline of *P. viticola* to CYA, with an EC_50_ value of 0.42 ± 0.05 μg/mL, and systematically clarify its resistance status in major grape-producing areas of China. Overall, *P. viticola* exhibited a high level of sensitivity to CYA, with a resistance frequency of only 28.47%, and the majority of resistant strains were classified as low-resistance isolates. This relatively low resistance frequency is likely attributable to the prevalent application of CYA in combination with therapeutic fungicides in agricultural production, rather than its sole use as a single agent. Currently, there are limited reports on CYA resistance worldwide, and most of these studies have focused on *Phytophthora* spp. Previous studies have indicated that the resistance of *Phytophthora* to CYA exhibits an increasing trend [33,34,48], which is consistent with the resistance trend observed in *P. viticola*. Notably, relatively high resistance frequencies, including a certain proportion of moderate and high-resistance strains, have been detected in regions such as Zhijiang and Gongan in China, indicating a potential risk of *P. viticola* developing resistance to CYA. Therefore, it is crucial to strengthen long-term resistance monitoring and implement rational fungicide rotation strategies in subsequent agricultural practices. Recent studies have reported that CYA resistance in French *P. viticola* populations is associated with mutations in the cytb gene [36]; future work will verify this mechanism in domestic strains and develop rapid detection techniques to support CYA resistance management and grape downy mildew control in China.

In addition to single-fungicide resistance, the potential emergence of multi-resistance should be considered within an evolutionary framework. Although only a weak but statistically significant correlation was detected between CYM and CYA sensitivities, and no strong cross-resistance was evident, the coexistence of isolates resistant to both fungicides indicates that resistance traits may accumulate within regional populations under sustained selection pressure. Such patterns are consistent with a stepwise selection model, in which independent resistance mutations arise and are sequentially enriched through repeated fungicide exposure.

Fitness changes in fungicide-resistant pathogen populations are critical to understanding field resistance dynamics, as resistance mutations inherently incur fitness costs as an evolutionary trade-off [49]. Here, we found that *P. viticola* resistant strains to those three fungicides (MET, CYM, and CYA) did not exhibit significant overall fitness disadvantages. Notably, metalaxyl-resistant strains showed significantly enhanced spore production compared to sensitive strains, while no statistical differences were observed in infectivity, lesion area, or comprehensive fitness index between resistant and sensitive strains for all three fungicides. This result is consistent with the findings of Genet and Jaworska [31] that there was no significant difference in fitness between resistant and susceptible strains of *P. viticola* to cymoxanil. Similar results were also observed when comparing the fitness of *P. viticola* resistant and susceptible strains to other fungicides, e.g., dimethomorph and azoxystrobin [50,51]; however, some azoxystrobin-resistant strains exhibited a fitness cost [52]. This may be attributed, on the one hand, to the differences in the modes of action of different fungicides, and on the other hand, to the fact that laboratory conditions may be more conducive to the growth of the pathogen. Therefore, additional field experiments are required for further verification in subsequent studies.

As a major global grape producer, sustainable control of grape downy mildew is critical for industrial development. Previous data on *P. viticola* resistance to MET, CYM, and CYA in China were scarce. This study systematically investigated resistance status in 9 major producing regions, established CYM and CYA sensitivity baselines, and clarified resistance distribution, cross-resistance relationships, and resistant strain fitness characteristics, filling relevant research gaps. Targeted control suggestions based on the results are as follows: (1) Regional precision application: Develop differentiated schemes based on local resistance. Use MET rationally in sensitive regions (e.g., Qingxu, Harbin) and prioritize CYA in sensitive regions (e.g., Langfang, Xing’an); avoid sole use in high-resistance regions. (2) Scientific rotation: Alternate CYM and CYA (no obvious cross-resistance) and rotate with MET (sensitive regions) or other mechanism fungicides to avoid long-term sole use. (3) Strengthen resistance monitoring: Establish a long-term system to track resistance changes, update application strategies in a timely manner for early detection and control. (4) Promote integrated control: Combine disease-resistant varieties, agronomic measures, and biological control to reduce chemical pesticide dependence and resistance pressure.

## Figures and Tables

**Table 1:** Information of *P. viticola* isolates used in this study.

Province	Collecting Sites	No. of Isolates for MET Sensitivity ^1^	No. of Isolates for CYM Sensitivity ^2^	No. of Isolates for CYA Sensitivity ^3^
Guangxi	Ziyuan	10	10	10
Yunnan	Binchuan	27	11	11
Hunan	Zhijiang	24	15	15
Hubei	Gong’an	50	58	25
Jiangsu	Jurong	16	17	17
Shandong	Yantai	50	8	8
Shanxi	Qingxu	20	31	31
Hebei	Langfang	26	10	10
Heilongjiang	Harbin	10	10	10
Total		233	170	137

^1^ MET: metalaxyl; ^2^ CYM: cymoxanil; ^3^ CYA: cyazofamid.

**Table 2:** Distribution of EC_50_ values of *P. viticola* to cymoxanil and cyazofamid.

Fungicide	Number of Isolates	EC_50_ (μg/mL)			
		Range	Mean	Standard Error	Variation Factor ^1^
Cymoxanil	170	0.01–691.39	36.44	7.38	69,139
Cyazofamid	137	0.01–60.39	2.15	0.56	6039

^1^ Variation factor: the highest EC_50_ value divided by the lowest EC_50_ value.

## Data Availability

The original contributions presented in the study are included in the article, further inquiries can be directed to the corresponding authors.

## Supplementary Materials

The following supporting information can be downloaded at: https://www.mdpi.com/article/10.3390/jof12030180/s1, Figure S1: Resistance levels of *P. viticola* from different regions to three fungicides (A) Metalaxyl; (B) Cymoxanil; (C) Cyazofamid (S = Sensitive, R = Resistant, LR = Low resistant, MR = Moderately resistant, HR = Highly resistant). Table S1: Resistance frequency of *P. viticola* to Metalaxyl, cymoxanil and cyazofamid. Table S2: Summary of *P. viticola* isolate resistance to both CYM and CYA.
