# Supplementary material for: Resistance Characterization of Plasmopara viticola to Metalaxyl, Cymoxanil, and Cyazofamid in China
Source: J Fungi (Basel). 2026 Mar 3;12(3):180. doi: 10.3390/jof12030180 (PMC13027896; doi:10.3390/jof12030180)
Supplement: Supplementary file 1 [file jof-12-00180-s001.zip › Tables S1 and S2.pdf]

Supplementary Tables

Table S1 Resistance frequency of *P. viticola* to Metalaxyl, cymoxanil and cyazofamid

Fungicide	Number of isolates	S	LR	MR	HR
Metalaxyl	233	106 (45.49%)	78 (33.48%)	49 (21.03%)	0
Cymoxanil	170	120 (70.59%)	36 (21.18%)	14 (8.24%)	0
Cyazofamid	137	98 (71.53%)	25 (18.25%)	13 (9.49%)	1(0.73%)

Note: S = sensitive, LR = low-resistant, MR = moderate-resistant, HR = high-resistant

Table S2 Summary of *P. viticola* isolate resistance to both CYM and CYA

Isolates	CYM	CYA
BC18-39	MR	MR
GA18-200	LR	LR
GA18-204	LR	LR
GA18-207	LR	LR
GA18-211	LR	LR
GA18-219	LR	MR
GA18-7	LR	MR
GXZY18-4	LR	LR
HLJ18-5-1	LR	LR
HuN18-14	LR	MR
HuN18-26	LR	MR
JR-18-1	LR	LR
JR-18-5	LR	LR
