# Supplementary figures and images for: Resistance Characterization of Plasmopara viticola to Metalaxyl, Cymoxanil, and Cyazofamid in China
Source: J Fungi (Basel). 2026 Mar 3;12(3):180. doi: 10.3390/jof12030180 (PMC13027896; doi:10.3390/jof12030180)

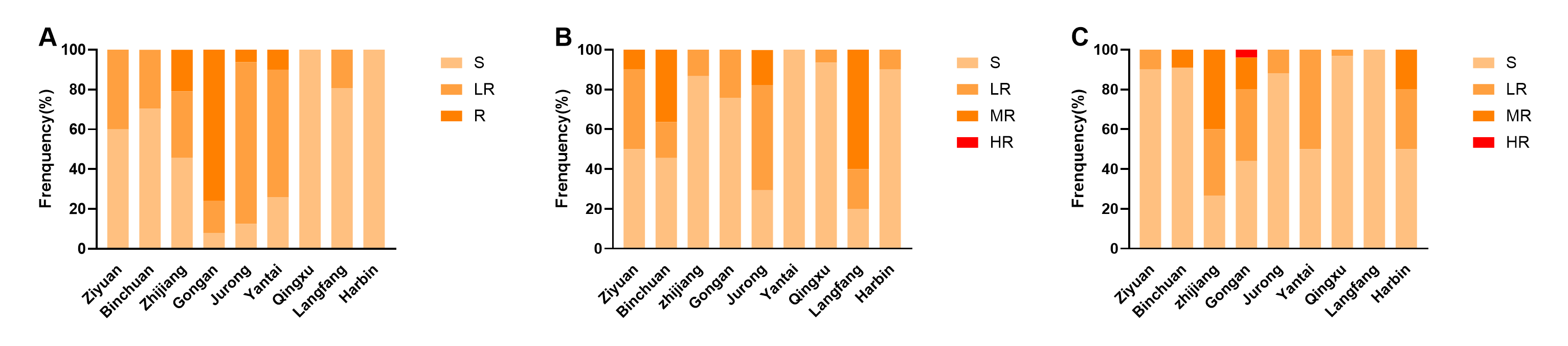

Supplement: Supplementary file 1 [file jof-12-00180-s001.zip › Figure S1.tif]
